# Echocardiographic findings in infants with presumed congenital Zika syndrome: Retrospective case series study

**DOI:** 10.1371/journal.pone.0175065

**Published:** 2017-04-20

**Authors:** Danielle Di Cavalcanti, Lucas V. Alves, Geraldo J. Furtado, Cleusa C. Santos, Fabiana G. Feitosa, Maria C. Ribeiro, Paulo Menge, Izabelle M. Lira, Joao G. Alves

**Affiliations:** 1 Department of Pediatrics, Instituto de Medicina Integral Prof. Fernando Figueira (IMIP), Recife, Pernambuco, Brazil; 2 Department of Pediatric Cardiology, Instituto de Medicina Integral Prof. Fernando Figueira (IMIP), Recife, Pernambuco, Brazil; Hospital Authority, CHINA

## Abstract

**Objective:**

To report the echocardiographic evaluation of 103 infants with presumed congenital Zika syndrome.

**Methods:**

An observational retrospective study was performed at Instituto de Medicina Integral Prof. Fernando Figueira (IMIP), Recife, Brazil. 103 infants with presumed congenital Zika syndrome. All infants had microcephaly and head computed tomography findings compatible with congenital Zika syndrome. Zika IgM antibody was detected in cerebrospinal fluid samples of 23 infants. In 80 infants, the test was not performed because it was not available at that time. All infants had negative serology for HIV, syphilis, rubella, cytomegalovirus and toxoplasmosis. A complete transthoracic two-dimensional, M-mode, continuous wave and pulsed wave Doppler and color Doppler echocardiographic (PHILIPS HD11XE or HD15) examination was performed on all infants.

**Results:**

14/103 (13.5%) echocardiograms were compatible with congenital heart disease: 5 with an ostium secundum atrial septal defect, 8 had a hemodynamically insignificant small apical muscular ventricular septal defect and one infant with dyspnea had a large membranous ventricular septal defect. The echocardiograms considered normal included 45 infants with a persistent foramen ovale and 16 with a minimum patent ductus arteriosus.

**Conclusions:**

Preliminarily this study suggests that congenital Zika syndrome may be associated with an increase prevalence of congenital heart disease. However the types of defects noted were septal defects, a proportion of which would not be hemodynamically significant.

## Introduction

An outbreak of Zika virus infection associated with congenital microcephaly has been reported in Brazil [1]. This association between Zika virus infection during pregnancy and microcephaly was initially prompted by Brazilian Ministry of Health in November 2015 [2] and in February 2016 the World Health Organization (WHO) announced this association as a Public Health Emergency of International Concern [3]. This association was finally confirmed by the Centers for Disease Control and Prevention (CDC) in April 2016 based on reports on detection of Zika viral nucleic acids in fetus, abnormal growth of infected neural stem cells and epidemiological studies [4].

Recently a presumed congenital Zika syndrome was described [5] —[6]. The main feature of this syndrome is the nervous system involvement: microcephaly, facial disproportionality, hypertonia, hyperreflexia, irritability and abnormal neuroimages–calcifications/ ventriculomegaly. Hearing and visual abnormalities may be present. As Zika virus infection appears to share many similarities to pre-natal rubella virus infection leading to serious birth defects, other organs or systems may be affected. Our aim was to report the echocardiographic evaluation of 103 infants (53 female) born in Brazil.

## Material and methods

### Study design, setting and study population

This was an observational retrospective study performed at Instituto de Medicina Integral Prof. Fernando Figueira (IMIP) Brazil from September 2015 to March 2016. We described the echocardiographic findings in 103 infants with presumed congenital Zika syndrome born between August 2015 and March 2016. During this same period 3,440 children were born at IMIP and 178 had microcephaly.

### Ethics statement

This study was approved by IMIP’s Ethics Committee in Research (n°2671–16). and all mothers of participants signed an informed consent after a full explanation of the purpose and nature of all procedures used.

### Inclusion criteria

All infants met clinical criterial for presumed congenital Zika syndrome, according to the Brazilian Ministry of Health recommendations (http://www.saude.go.gov.br/public/media/ZgUINSpZiwmbr3/10100011602222060026.pdf 2016), were included: a head circumference below the third percentile for gestational age and gender by the Fenton growth chart [7] and a head computed tomography with the following findings: intracranial calcifications, ventriculomegaly and global hypogyration of the cerebral cortex and on maternal manifestations suggestive of Zika virus infection. Positive serology (IgG and IgM) for HIV, syphilis, rubella, cytomegalovirus or toxoplasmosis was considered as an exclusion criteria.

Zika virus IgM antibody capture enzyme-linked immunosorbent assay was performed in cerebrospinal fluid samples. However this exam was not performed in all infants because this test was not available at that time.

### Cardiac evaluation

A clinical cardiologic evaluation was performed in all studied infants by a pediatrician and a pediatric cardiologist. A complete transthoracic two-dimensional, M-mode, continuous wave and pulsed wave Doppler and color Doppler echocardiographic (PHILIPS HD11XE or HD15) examination was performed on all infants by an experienced pediatric cardiologist. Two-dimensional and color Doppler imaging were taken from the suprasternal, parasternal, subxiphoid, apical and, if necessary, modified views.

Congenital heart disease (CHD) was defined as a gross structural abnormality of the heart [8]. A patent ductus arteriosus (PDA) in the first 1 month of life was not considered to be cardiac anomalie [9]—[10]—[11].

A patente foramen ovale (PFO) in the typical position, and size < 3 mm, were considered normal at this age.

### Statistical analysis

CHD prevalence was based on the number of live births with CHD divided by all 103 newborns with congenital Zika syndrome. The data were presented as mean with 95% confidence interval and percentage.

## Results

Some characteristics of these children are shown at Table 1. All infants showed calcifications, cortical hypogyration, ventriculomegaly or white-matter abnormalities on CT. Zika virus IgM antibody capture enzyme-linked immunosorbent assay was detected in cerebrospinal fluid samples of 23 infants; 80 infants did not perform this exam because this test was not available at that time.

**Table 1:** Some characteristics of the 103 infants with presumed congenital Zika syndrome.

Characteristic	n°	% or 95% CI
**Maternal rash during pregnancy**	**49**	**47.5**
**Preterm**	**12**	**11.6**
**Birth weight (g) mean**	**2,653**	**2.573–2.733**
**Birth height (cm) mean**	**45.7**	**45.2–46.2**
**Head circumference (cm) mean**	**28.8**	**28.4–39.3**
**Congenital heart disease**	**14**	**14.5**
**Atrial septal defect**	**5**	**5.1**
**Ventricular septal defect**	**9**	**8.7**

A clinical cardiologic evaluation was normal, except for one infant with dyspnea. Echocardiographic images were obtained at a mean age of 58 days (2 to 150). 14/103 (13.5%) echocardiograms were compatible with CHD; prevalence of 135/1,000: 5 with an ostium secundum atrial septal defect (all ≤ 4 mm and ≥ 3 mm), 8 had a small apical muscular ventricular septal defect hemodynamically insignificant and one infant with dyspnea had a large membranous ventricular septal defect. The echocardiograms considered normal included 45 newborns with a persistent foramen ovale and 16 with a minimum patent ductus arteriosus, all within the first 28 days of life.

Cardiac function and wall thickness were considered normal except an infant with a large VSD. Effusions, valvar abnormalities, coronary artery anomalies or dilatation, thrombosis or vegetations were not detected in any newborn/infant.

## Discussion

Our echocardiographic findings in infants with presumed congenital Zika syndrome were almost three times the expected rate of CHD in a general population less than a year of age, at 135 per 1000 live births. The prevalence of CHD may range from 4 to 50 per 1000 live births depending on the diagnostic method used and subject´s age [12]. CHD prevalence is higher, about 50/1.000, using echocardiography method and during the first months of life as we did. Echocardiography method may detect asymptomatic and small cardiac abnormalities and some CHD may spontaneously improve during the first months of life. Children with hemodynamically insignificant congenital cardiac lesions are usually asymptomatic and have a benign course with negligible risk to the child. [13].

Almost all infants studied showed a minimal septal defect and only one infant had dyspnea and showed a large membranous VSD. All atrial septal defects had < 4 mm in diameter which are very likely to decrease in size or completely close on follow-up. However a small or moderate ventricular septal defect may still be significant if it causes aortic insufficiency or outflow tract obstruction as in the case of double chamber right ventricle. The size alone does not necessarily mean it is not hemodynamically significant. Furthermore it is not known if Zika exposed infants have the same spontaneous regression or closure rate of minor CHD as described in a normal population. Infants with small muscular VSDs might be easily missed without colour Doppler echocardiography. Muscular VSD closed earlier than perimembranous VSD and a recently review [14] observed that all muscular VSD < 3.0 mm closed before the age of 3 years old. This corroborates our finding that only one of the 103 infants studied had signs and symptomns of CHD.

This is the first time that CHD was assessed in infants with presumed congenital Zika syndrome. Currently there are no reports or autopsy evidence of any cardiac defects associated with presumed congenital Zika syndrome. Many others malformations have been described in the presumed congenital Zika syndrome, especially the central nervous system manifestations. As congenital Zika syndrome has many similarities with congenital Rubella syndrome it would be important to rule out cardiac involvement. This study seems to add a new contribution by showing apparently an increased rate of CHD in infants with congenital Zika infection. As we are reporting preliminary findings in a few infants, our results need to be confirmed by others studies.

Currently studies on the relationship between Zika virus infection and heart involvment are lacking. Other Flavivirus such as dengue virus have been associated with myocarditis and pericarditis [15]–[16]. We did not find in our studied infants evidence of pericardium or myocardium involvement in echocardiography, besides these infants did not have any clinical manifestation. Recently Krittanawong et al [17] highlight the potential cardiovascular complications of Zika virus infection and suggested a prompt cardiovascular screening in suspect cases of Zika virus infection.

Our observational study has some limitations. At first we could not make serological diagnosis in all studied infants; 80 infants did not perform this test because this exam was not available at that time. However Zika virus IgM antibody was detected in cerebrospinal fluid samples of 23 infants and all infants had microcephaly and neuroimages alterations compatible with congenital Zika infection. Besides the current outbreak of microcephaly associated with Zika infection during pregnancy in Brazil especially in the Northeast region where IMIP is located makes the diagnosis of congenital Zika infection quite plausible. Second, stillbirths were not studied and this exclusion may underestimate the CHD prevalence in congenital Zika syndrome.

## Conclusion

These preliminary findings suggest a high prevalence of CHD in presumed congenital Zika syndrome. All the studied infants are being followed with subsequent echocardiogram at a year of age to determine spontaneous regression of the minor cardiac defects detected.

## Supporting information

S1 File Compatible echocardiograms with congenital heart disease. (DOCX)
