# Supplementary material for: Echocardiographic findings in infants with presumed congenital Zika syndrome: Retrospective case series study
Source: PLoS One. 2017 Apr 20;12(4):e0175065. doi: 10.1371/journal.pone.0175065 (PMC5398518; doi:10.1371/journal.pone.0175065)
Supplement: S1 File — (DOCX) [file pone.0175065.s001.docx]

Patient:

Age: 02 months

Weigth: 4,8 Kg

Heigt: 0,57 m Body area: 0,35 m2

______________________________________________________________________________

1. * LINEA MEASURESRES *

LEFT VENTRICLE

Transversal axis normal values

Final diastolic diameter final.: 18 mm (29+-08 mm)

Final systolic diameterco final..: 12 mm (--+- - mm)

Interventricular sept....: 4 mm (05+-02 mm)

Posterior wall..........: 3 mm (04+-02 mm)

Longitudinal axis

Final diastolic diameter: 32 mm (--+- - mm)

Final systolic diameter..: 29 mm (--+- - mm)

Anterior wall ...........: 3 mm (--+- - mm)

AORTA

Valvar ring ...............: 8 mm (--+- - mm)

Final systolic diameter..: 9 mm (17+-04 mm)

LEFT ATRIUM

Final systolic diameter,.: 12 mm (19+-06 mm)

Final diastolic volume...: 11 cm³/m (16-34 cm³/m)

RIGHT VENTRICLE

Final diastolic diameter .: 8 mm (09+-05 mm)

2. * VOLUME, FUNCTION AND RELATIONS *

LEFT VENTRICLE

Final diastolic volume ...: 5 ml *

Final systolic volume....: 2 ml *

Ventricle Mass E.......: 6 g *

% shortening ........: 33 (36+-6%)

Ejection fraction ..........: 0,60 ( 0, 60 )

RELATION LA/Ao.............: 1,33 (< 1,5 )

RELATION SIV/PPVE..........: 1,33 (< 1,5 )

RELATION VDF/MVE...........: 0,86 (> 0,7 )

MASS INDICE LV.....: 18,1 g/m2

* Calculated by ELIPSÓIDE BIPLANAR

3. * HEART DEBIT *

Cardiac rate .......: 180 bpm

Volume ejected LV......: 4,59 ml *

Left heart debit ..: 0,83 l/m

Volume ejected RV......: 5,14 ml *

Rigth heart debit ...: 0,93 l/m

Qp/Qs.....................: 1,12

* Calculated by DOPPLER PULS/CONT

**Date**.:

Exam no.:

4. * EXAM DESCRIPTION *

Ecocardiogram performed realizado nas posições paraesternal E., apical E. e subcostal.

5. * VALVES AV *

Normais.

6. * SIGMOIDS VALVES *

Normals.

7. * RIGHT CAVITIES *

8. * LEFT CAVITIES *

9. * PERICARDIUM *

Normal.

10.* * PULSTABLE / CONTINUOUS AND TISSULAR DOPPLER *

Minimum flow AE-> AD in SIA OII.

11. * COLOR FLOW MAPPING *

Minimum flow AE-> AD in SIA OII. Minimum flow printing To-> APE.

12. * COMMENTS AND CONCLUSION

Situs solitus with levocardia. AV and VA concordance.

Minimal continuity solution at the interatrial septum in ostium secundum (3mm) with minimum flow E-> D without signs of repercussion and function

Normal biventricular. We can not rule out the possibility of minimum channel

Arterial hypertension.

Patient:

Age: 2 months

Weigth: 3,8 Kg

Heigth: 54 m Body area: 27,05 m2

______________________________________________________________________________

1. * LINEAR MEASURES *

LEFT VENTRICLE

Transversal axis.

Final Diastolic Diameter: 22 mm (46 + -06 mm)

Final systolic diameter: 14 mm (29 + -05 mm)

Interventricular septum: 4 mm (09 + -02 mm)

Rear wall ..........: 3 mm (07 + -02 mm)

Longitudinal Axis

Final Diastolic Diameter: 33 mm (71 + -11 mm)

Final systolic diameter: 28 mm (63 + -13 mm)

Front wall ...........: 3 mm (09 + -02 mm)

AORTA

Valve ring ...............: 7 mm (20 + -03 mm)

Final systolic diameter: 9 mm (24 + -04 mm)

LEFT ATTRACTION

Final systolic diameter: 10 mm (30 + -06 mm)

Final diastolic volume ...: 0 cm³ / m (16-34 cm³ / m)

RIGHT VENTRICULAR

Final Diastolic Diameter: 10 mm (19 + -06 mm)

2. * VOLUMES, FUNCTION AND RELATIONSHIPS *

LEFT VENTRICLE

Final Diastolic Volume ...: 8 ml *

Final systolic volume ....: 3 ml *

Ventricular mass E .......: 8 g *

% Shortening ........: 36 (36 + -6%)

Ejection fraction ..........: 0.66 (0.60)

AE / Ao Ratio: 1.11 (<1.5)

SIV / PPVE RELATION ..........: 1.33 (<1.5)

VDF / MVE ratio ...........: 1.06 (> 0.7)

VE mass index: 0.3 g / m2

* Calculated by ELIPHID BIPLANAR

3. * HEART DEBIT *

Heart rate ....... 115 bpm

VE ejected volume ......: 4.55 ml *

Left heart defect: 0.52 l / m

VD ejected volume ......: 4.02 ml *

Right heart rate ...: 0.46 l / m

Qp / Qs .....................: 0.88

* Calculation done by DOPPLER PULS / CONT

Date.:

Exam No .:

______________________________________________________________________________

4. * DESCRIPTION OF THE EXAM *

Echocardiogram performed in parasternal E., apical E. and subcostal positions.

5. * AV VALVES *

Normal

6. * SIGMON VALVES *

Normal

7. * RIGHT CAVITIES *

8. * LEFT CAVITIES *

9. * PERICÁRDIO *

Normal.

10. * PULSTABLE / CONTINUOUS AND TISSULAR DOPPLER *

Flow AE-> AD in SIA OII and minimal flow Ao-> APE.

11. * COLOR FLOW MAPPING *

Flow AE-> AD in SIA OII and minimum flow Ao-> APE.

12. * COMMENTS AND CONCLUSIONS *

Situs solitus with levocardia. AV and VA concordance.

VCI and normal VCS. Atrium D. and ventricle D of conserved dimensions with

Normal RV. A. PULMONARY normal. APE and normal APD.

Pulmonary veins with habitual implantation. Atrium E. and ventricle E. of dimensions

With normal LV function. Ascending aorta, crossa and descending

normal. SIA with small continuity solution in ostium secundum (3mm).

SIV intact. LV walls normal. CD and CE of usual origin.

Continuity solution at the interatrial septum in ostium secundum (2.5 to 3mm ) With E> D flow, minimal patent ductus arteriosus with Ao-> APE

With no signs of repercussion and normal biventricular function.

Patient:

Idade...: 03 months

Peso....: 5,3 Kg

Altura..: 0,57 m Body área: 0,36 m2

______________________________________________________________________________

1. * LINEAR MEASURES *

LEFT VENTRICLE

Transversal Axis Normal Values

Final Diastolic Diameter: 19 mm (29 + -08 mm)

Final systolic diameter: 13 mm (- + - - mm)

Interventricular septum: 5 mm (05 + -02 mm)

Rear wall ..........: 4 mm (04 + -02 mm)

Longitudinal Axis

Final Diastolic Diameter: 34 mm (- + - - mm)

Final systolic diameter: 31 mm (- + - - mm)

Front wall ...........: 3 mm (- + - - mm)

AORTA

Valve ring ...............: 9 mm (- + - - mm)

Final systolic diameter: 11 mm (17 + -04 mm)

LEFT ATTRACTION

Final systolic diameter: 13 mm (19 + -06 mm)

Final diastolic volume ...: 8 cm³ / m (16-34 cm³ / m)

RIGHT VENTRICULAR

Final Diastolic Diameter: 11 mm (09 + -05 mm)

2. * VOLUMES, FUNCTION AND RELATIONSHIPS *

LEFT VENTRICLE

Final Diastolic Volume ...: 6 ml *

Final systolic volume ....: 3 ml *

Ventricular mass E .......: 9 g *

% Shortening ........: 32 (36 + -6%)

Ejection fraction ..........: 0.57 (0.60)

AE / Ao Ratio: 1.18 (<1.5)

SIV / PPVE RELATION ..........: 1.25 (<1.5)

VDF / MVE ratio ...........: 0.70 (> 0.7)

VE mass index: 25.8 g / m2

* Calculated by ELIPHID BIPLANAR

3. * HEART DEBIT *

Heart rate ....... 150 bpm

VE ejected volume ......: 6.31 ml *

Left heart defect: 0.95 l / m

VD ejected volume ......: 5.78 ml *

Right heart rate ...: 0.87 l / m

Qp / Qs .....................: 0.92

* Calculation done by DOPPLER PULS / CONT

Date.:

Exam No .:

______________________________________________________________________________

4. * DESCRIPTION OF THE EXAM *

Echocardiogram performed in parasternal E., apical E. and subcostal positions.

5. * AV VALVES *

Normal

6. * SIGMON VALVES *

Normal

7. * RIGHT CAVITIES *

8. * LEFT CAVITIES *

9. * PERICÁRDIO *

Normal.

10. * PULSTABLE / CONTINUOUS AND TISSULAR DOPPLER *

Flow AE-> AD in SIA OII.

11. * COLOR FLOW MAPPING *

Flow AE-> AD in SIA OII.

12. * COMMENTS AND CONCLUSIONS *

Situs solitus with levocardia. AV and VA concordance.

VCI and normal VCS. Atrium D. and ventricle D of conserved dimensions with

Normal RV. A. PULMONARY normal. APE and normal APD.

Pulmonary veins with habitual implantation. Atrium E. and ventricle E. of dimensions

With normal LV function. Ascending aorta, crossa and descending

normal. SIA with continuity solution in ostium secundum (4mm). SIV intact.

LV walls normal. CD and CE of usual origin.

Interatrial septum continuity solution in ostium secundum (4mm) with flow E> D Without signs of repercussion and normal biventricular function.

Patient:

Age: 02 months

Weigth: 4,4 Kg

Heigth: 0,51 m Body area: 0,31 m2

______________________________________________________________________________

1. * LINEAR MEASURES *

LEFT VENTRICLE

Transversal Axis Normal Values

Final Diastolic Diameter: 19 mm (29 + -08 mm)

Final systolic diameter: 12 mm (- + - - mm)

Interventricular septum: 4 mm (05 + -02 mm)

Rear wall ..........: 3 mm (04 + -02 mm)

Longitudinal Axis

Final Diastolic Diameter: 33 mm (- + - - mm)

Final systolic diameter: 30 mm (- + - - mm)

Front wall ...........: 3 mm (- + - - mm)

AORTA

Valve ring ...............: 7 mm (- + - - mm)

Final systolic diameter: 8 mm (17 + -04 mm)

LEFT ATTRACTION

Final systolic diameter: 13 mm (19 + -06 mm)

Final diastolic volume ...: 18 cm³ / m (16-34 cm³ / m)

RIGHT VENTRICULAR

Final Diastolic Diameter: 9 mm (09 + -05 mm)

2. * VOLUMES, FUNCTION AND RELATIONSHIPS *

LEFT VENTRICLE

Final Diastolic Volume ...: 6 ml *

Final systolic volume ....: 2 ml *

Ventricular mass E .......: 7 g *

% Shortening ........: 37 (36 + -6%)

Ejection fraction ..........: 0.64 (0.60)

AE / Ao Ratio: 1.63 (<1.5)

SIV / PPVE RELATION ..........: 1.33 (<1.5)

VDF / MVE Ratio ...........: 0.91 (> 0.7)

VE mass index: 21.7 g / m2

* Calculated by ELIPHID BIPLANAR

3. * HEART DEBiT *

Heart rate ....... 154 bpm

VE ejected volume ......: 4.64 ml *

Left heart defect: 0.71 l / m

VD ejected volume ......: 6,31 ml *

Right heart rate ...: 0.97 l / m

Qp / Qs .....................: 1.36

* Calculation done by DOPPLER PULS / CONT

Date.:

Exam No .:

4. * DESCRIPTION OF THE EXAM *

Echocardiogram performed in parasternal E., apical E. and subcostal positions.

5. * AV VALVES *

Normal

6. * SIGMON VALVES *

Normal

7. * RIGHT CAVITIES *

8. * LEFT CAVITIES *

9. * PERICÁRDIO *

Normal.

10. * PULSTABLE / CONTINUOUS AND TISSULAR DOPPLER *

Flow AE-> AD in SIA OII.

11. * COLOR FLOW MAPPING *

Flow AE-> AD in SIA OII.

12. * COMMENTS AND CONCLUSIONS *

Situs solitus with levocardia. AV and VA concordance.

VCI and normal VCS. Atrium D. and ventricle D of conserved dimensions with

Normal RV. A. PULMONARY normal. APE and normal APD.

Pulmonary veins with habitual implantation. Atrium E. and ventricle E. of dimensions

With normal LV function. Ascending aorta, crossa and descending

normal. SIA with small continuity solution in high portion of ostium secundum

(2.4mm). SIV intact. LV walls normal. CD and CE of usual origin.

Small atrial septal defect in ostium secundum (3 mm) with

E> D flow without signs of repercussion and normal biventricular function.

Patient: Age: 01 month

Weigth: 3,8 Kg

Heigth: 0,5 m Body area: 0,30 m2

______________________________________________________________________________

1. * LINEAR MEASURES *

LEFT VENTRICLE

Transversal Axis Normal Values

Final Diastolic Diameter: 18 mm (29 + -08 mm)

Final systolic diameter: 11 mm (- + - - mm)

Interventricular septum: 3 mm (05 + -02 mm)

Rear wall ..........: 3 mm (04 + -02 mm)

Longitudinal Axis

Final Diastolic Diameter: 28 mm (- + - - mm)

Final systolic diameter: 26 mm (- + - - mm)

Front wall ...........: 3 mm (- + - - mm)

AORTA

Valve ring ...............: 6 mm (- + - - mm)

Final systolic diameter: 10 mm (17 + -04 mm)

LEFT ATTRACTION

Final systolic diameter: 13 mm (19 + -06 mm)

Final diastolic volume ...: 7 cm³ / m (16-34 cm³ / m)

RIGHT VENTRICULAR

Final Diastolic Diameter: 7 mm (09 + -05 mm)

2. * VOLUMES, FUNCTION AND RELATIONSHIPS *

LEFT VENTRICLE

Final Diastolic Volume ...: 5 ml *

Final systolic volume ....: 2 ml *

Ventricular mass E .......: 5 g *

% Shortening ........: 39 (36 + -6%)

Ejection fraction ..........: 0.65 (0.60)

AE / Ao Ratio: 1.30 (<1.5)

SIV / PPVE RELATION ..........: 1.00 (<1.5)

VDF / MVE ratio ...........: 0.98 (> 0.7)

VE mass index: 16.0 g / m2

* Calculated by ELIPHID BIPLANAR

3. * HEART DEBATE *

Heart rate ....... 200 bpm

VE ejected volume ......: 3.59 ml *

Left heart defect: 0.72 l / m

VD ejected volume ......: 2.54 ml *

Right heart rate ...: 0.51 l / m

Qp / Qs ...: 0.71

* Calculation done by DOPPLER PULS / CONT

Date.:

Exam No .:

______________________________________________________________________________

4. * DESCRIPTION OF THE EXAM *

Echocardiogram performed in parasternal E., apical E. and subcostal positions.

5. * AV VALVES *

Normal

6. * SIGMON VALVES *

Normal

7. * RIGHT CAVITIES *

8. * LEFT CAVITIES *

9. * PERICÁRDIO *

Normal.

10. * PULSTABLE / CONTINUOUS AND TISSULAR DOPPLER *

Flow VE-> VD in SIV.

11. * COLOR FLOW MAPPING *

Flow VE-> VD in SIV. Continuous flow printing To-> APE.

12. * COMMENTS AND CONCLUSIONS *

Situs solitus with levocardia. AV and VA concordance.

VCI and normal VCS. Atrium D. and ventricle D of conserved dimensions with

Normal RV. A. PULMONARY normal. APE and normal APD.

Pulmonary veins with habitual implantation. Atrium E. and ventricle E. of dimensions

With normal LV function. Ascending aorta, crossa and descending

normal. SIA intact. SIV with small solution of apical muscle continuity.

LV walls normal. CD and CE of usual origin.

Congenital cardiomyopathy type apical muscular interventricular communication with flow

E> D without signs of repercussion and normal biventricular function. Channel Print

Arterial

Patient:

Age: Idade...: 02 months

Weigth: 4,4 Kg

Heigth: 0,54 m Área Corporal: 0,33 m2

______________________________________________________________________________

1. * LINEAR MEASURES *

LEFT VENTRICLE

Transversal Axis Normal Values

Final Diastolic Diameter: 23 mm (29 + -08 mm)

Final systolic diameter: 13 mm (- + - - mm)

Interventricular septum: 4 mm (05 + -02 mm)

Rear wall ..........: 3 mm (04 + -02 mm)

Longitudinal Axis

Final Diastolic Diameter: 32 mm (- + - - mm)

Final systolic diameter: 30 mm (- + - - mm)

Front wall ...........: 3 mm (- + - - mm)

AORTA

Valve ring ...............: 8 mm (- + - - mm)

Final systolic diameter: 9 mm (17 + -04 mm)

LEFT ATTRACTION

Final systolic diameter: 14 mm (19 + -06 mm)

Final diastolic volume ...: 16 cm³ / m (16-34 cm³ / m)

RIGHT VENTRICULAR

Final Diastolic Diameter: 10 mm (09 + -05 mm)

2. * VOLUMES, FUNCTION AND RELATIONSHIPS *

LEFT VENTRICLE

Final diastolic volume ...: 9 ml *

Final systolic volume ....: 3 ml *

Ventricular mass E .......: 8 g *

% Shortening ........: 43 (36 + -6%)

Ejection fraction ..........: 0.70 (0.60)

AE / Ao Ratio: 1.56 (<1.5)

SIV / PPVE RELATION ..........: 1.33 (<1.5)

VDF / MVE ratio ...........: 1.11 (> 0.7)

VE mass index: 24.3 g / m2

* Calculated by ELIPHID BIPLANAR

3. * HEART DEBATE *

Heart rate ....... 128 bpm

VE ejected volume ......: 4.23 ml *

Left heart defect: 0.54 l / m

VD ejected volume ......: 5.02 ml *

Right heart rate ...: 0.64 l / m

Qp / Qs .....................: 1.18

* Calculation done by DOPPLER PULS / CONT

Date.:

Exam No .:

______________________________________________________________________________

4. * DESCRIPTION OF THE EXAM *

Echocardiogram performed in parasternal E., apical E. and subcostal positions.

5. * AV VALVES *

Normal

6. * SIGMON VALVES *

Normal

7. * RIGHT CAVITIES *

8. * LEFT CAVITIES *

9. * PERICÁRDIO *

Minimal diffuse laminar effusion without restriction signs.

10. * PULSTABLE / CONTINUOUS AND TISSULAR DOPPLER *

Flow VE-> RV in apical muscular SIV and minimal continuous flow Ao-> APE.

11. * COLOR FLOW MAPPING *

Flow VE-> RV in apical muscular SIV and minimal continuous flow Ao-> APE.

12. * COMMENTS AND CONCLUSIONS *

Situs solitus with levocardia. AV and VA concordance.

VCI and normal VCS. Atrium D. and ventricle D of conserved dimensions with

Normal RV. A. PULMONARY normal. APE and normal APD.

Pulmonary veins with habitual implantation. Atrium E. and ventricle E. of dimensions

With normal LV function. Ascending aorta, crossa and descending

normal. SIA intact. SIV with minimal solution of apical muscle continuity.

LV walls normal. CD and CE of usual origin.

Minimal apical muscular ventricular septal defect with minimal flow E-> D without

Signs of repercussion, minimal patent ductus arteriosus with E> D flow with no signs of

Repercussion and normal biventricular function. Presence of minimal pericardial effusion

Diffuse lamina with no signs of restriction.

**PEDIATRIC CARDIOLOGY**

**ECHOCARDIOGRAM**

|  | AGE: 2 days | GENDER: FEMALE | RG |
| --- | --- | --- | --- |
| PATIENT: | | | |

SEGMENTAL ANALYSIS

Situs solitus, levocardia and levoposite of the apex.

Normal systemic and pulmonary venous connections.

Concordant biventricular atrioventricular connection, two-valve mode.

Concordant biarterial ventriculo-arterial connection, two-valves mode

Interatrial septum with left foramen oval foramen with left-right flow to color flow mapping

Interventricular septal with two small apical muscular interventricular communications with left-right shunt to color flow mapping.

Closed arterial channel

LEFT CAVITIES:

Left atrium: Normal size

Left ventricle: Normal dimension. Normal myocardial thickness. Normal systolic function

RIGHT CAVITY:

Right atrium: Normal size.

Right ventricle: Normal size. Normal myocardial thickness. Normal systolic function

BASIC VESSELS:

Aorta: Normal size. Left aortic arch, unobstructed along its path.

Crowns of habitual origin.

Pulmonary artery: Main pulmonary artery and right and left pulmonary arteries of normal dimensions.

VALVAR APPLIANCES:

The cardiac valves have normal characteristics, as well as the analysis of their flow to the color flow mapping

PERICARDIUM:

Absence of pericardial effusion.

CONCLUSION:

Persistent oval foramen

Two small interventricular communications apical muscle

Preserved biventricular function.

**PEDIATRIC CARDIOLOGY**

**ECODOPPLER CARDIOGRAM TRANSTORACIC**

|  | AGE: 20 dias | GENDER: MALE | RG: |
| --- | --- | --- | --- |
| PATIENT: | | | |

SEGMENTAL ANALYSIS

Situs solitus, levocardia and levoposite of the apex.

Normal systemic and pulmonary venous connections.

Concordant biventricular atrioventricular connection, two-valves mode

Concordant biarterial ventriculo-arterial connection, two-valves mode

Integral atrial septal with patent foramen ovale with left-right flow to the color flow mapping in the atrial plane.

Presence of perimembranous interventricular communication of approximately 3.5 mm, partially covered by subtricuspid tissue. Left-right shunt by IVC with a VE-VD gradient of 60mmHg.

Closed arterial channel

LEFT CAVITIES:

Left atrium: Normal size.

Left ventricle: Normal dimension. Normal myocardial thickness. Normal systolic function

RIGHT CAVITY:

Right atrium: Normal size

Right ventricle: Normal size. Normal myocardial thickness. Normal systolic function

BASIC VESSELS:

Aorta: Aorta of normal size. Left aortic arch, unobstructed along its path.

Crowns of habitual origin.

Pulmonary artery: Main pulmonary artery and right and left pulmonary arteries of normal dimensions.

VALVAR APPLIANCES:

Aortic valve: Fine leaflets, preserved mobility and aperture

Pulmonary Valve: Fine leaflets, preserved mobility and aperture

Mitral Valve: Fine leaflets, preserved mobility and aperture

Tricuspid valve: Fine leaflets, preserved mobility and aperture.

PERICARDIUM:

Absence of pericardial effusion.

DOPPLER:

QP / QS = 2.4: 1

CONCLUSION:

Small perimembranous interventricular communication without hemodynamic repercussion

Persistent oval foramen

Preserved biventricular function.

**PEDIATRIC CARDIOLOGY**

**ECHOCARDIOGRAM**

|  | AGE: 8 days | GENDER: Female | RG: |
| --- | --- | --- | --- |
| PATIENT: | | | |

SEGMENTAL ANALYSIS

Situs solitus, levocardia and levoposite of the apex.

Normal systemic and pulmonary venous connections.

Concordant biventricular atrioventricular connection, two-valve mode.

Concurrent biarterial ventriculo-arterial connection, two-valve mode.

Interatrial septum with patent foramen ovale with left-right shunt.

Interventricular septal with presence of minimal apical muscular interventricular communication with left-right shunt.

Closed arterial channel

LEFT CAVITIES:

Left atrium: Normal size

Left ventricle: Normal dimension. Normal myocardial thickness. Normal systolic function

RIGHT CAVITY:

Right atrium: Normal size.

Right ventricle: Normal size. Normal myocardial thickness. Normal systolic function

BASIC VESSELS:

Aorta: Normal size. Left aortic arch, unobstructed along its path.

Crowns of habitual origin.

Pulmonary artery: Main pulmonary artery and right and left pulmonary arteries of normal dimensions.

VALVAR APPLIANCES:

Aortic valve: Fine leaflets, preserved mobility and aperture

Pulmonary Valve: Fine leaflets, preserved mobility and aperture. Displays discrete reflux.

Mitral Valve: Fine leaflets, preserved mobility and aperture

Tricuspid valve: Fine leaflets, preserved mobility and aperture.

PERICARDIUM:

Absence of pericardial effusion.

CONCLUSION:

Persistent oval foramen

Minimum apical muscular interventricular communication.

Preserved biventricular function.

**PEDIATRIC CARDIOLOGY**

**ECODOPPLER CARDIOGRAMA TRANSTORÁCICO**

|  | AG: 03 months | GENDER: MALE | RG: |
| --- | --- | --- | --- |
| PATIENT: | | | |

SEGMENTAL ANALYSIS

Situs solitus, levocardia and levoposite of the apex.

Normal systemic and pulmonary venous connections.

Concordant biventricular atrioventricular connection, two-valves mode

Concordant biarterial ventriculo-arterial connection, two-valves mode

Interatrial septum with patent foramen ovale. Shunt from left to right to color flow mapping in the atrial plane.

Presence of wide perimembranous ventricular septal defect with extension to the outlet of approximately 8.0 mm, presence of discrete posterior deviation of the infundibular septum. Shunt from left to right by IVC with a VE-VD gradient of 55 mmHg.

Closed arterial channel

LEFT CAVITIES:

Left atrium: Moderate dilatation.

Left ventricle: Moderate degree dilatation. Normal myocardial thickness. Normal systolic function

RIGHT CAVITY:

Right atrium: Discrete degree dilatation.

Right ventricle: Dilatation of discrete degree. Discreet hyperperfia. Normal systolic function

BASIC VESSELS:

Aorta: Dilation of the aortic root. Left aortic arch, unobstructed along its path.

Crowns of habitual origin.

Pulmonary artery: Main pulmonary artery and right pulmonary artery of normal dimensions. Discrete dilation of the left pulmonary artery.

VALVAR APPLIANCES:

Aortic valve: Fine leaflets, preserved mobility and aperture

Pulmonary valve: thickened leaflets, opening in dome, opening discretely diminished. Transvalvular turbulent flow with a maximum systolic gradient of 66 mmHg (gradient may be overestimated by the hyperflow caused by the VSD).

Mitral Valve: Fine leaflets, preserved mobility and aperture

Tricuspid valve: Fine leaflets, preserved mobility and aperture.

PERICARDIUM:

Absence of pericardial effusion.

CONCLUSION:

Wide perimembranous ventricular septal defect with extension to the outflow tract with hemodynamic repercussion

Patent oval foramen

Moderate pulmonary valve stenosis (gradient may be overestimated by hyperflow)

Preserved biventricular function

**PEDIATRIC CARDIOLOGY**

**ECHOCARDIOGRAM**

|  | AGE: 8 dias | GENDER: Female | RG |
| --- | --- | --- | --- |
| PATIENT: | | | |

SEGMENTAL ANALYSIS

Situs solitus, levocardia and levoposite of the apex.

Normal systemic and pulmonary venous connections.

Concordant biventricular atrioventricular connection, two-valve mode.

Concurrent biarterial ventriculo-arterial connection, two-valve mode.

Interatrial septum with patent foramen ovale with left-right shunt.

Interventricular septal with presence of minimal apical muscular interventricular communication with left-right shunt.

Closed arterial channel

LEFT CAVITIES:

Left atrium: Normal size

Left ventricle: Normal dimension. Normal myocardial thickness. Systolic function

normal

RIGHT CAVITY:

Right atrium: Normal size.

Right ventricle: Normal size. Normal myocardial thickness. Normal systolic function

BASIC VESSELS:

Aorta: Normal size. Left aortic arch, unobstructed along its path.

Crowns of habitual origin.

Pulmonary artery: Main pulmonary artery and right and left pulmonary arteries of normal dimensions.

VALVAR APPLIANCES:

Aortic valve: Fine leaflets, preserved mobility and aperture

Pulmonary Valve: Fine leaflets, preserved mobility and aperture. Displays discrete reflux.

Mitral Valve: Fine leaflets, preserved mobility and aperture

Tricuspid valve: Fine leaflets, preserved mobility and aperture.

PERICARDIUM:

Absence of pericardial effusion.

CONCLUSION:

Persistent oval foramen

Minimum apical muscular interventricular communication.

Preserved biventricular function

**PEDIATRIC CARDIOLOGY**

**ECHOCARDIOGRAM**

|  | AGE: 12 days | GENDER: male |  |
| --- | --- | --- | --- |
| PATIENT: | | | |

SEGMENTAL ANALYSIS

Situs solitus, levocardia and levoposite of the apex.

Normal systemic and pulmonary venous connections.

Concordant biventricular atrioventricular connection, two-valve mode.

Concurrent biarterial ventriculo-arterial connection, two-valve mode.

Interatrial septum with foramen oval foramen with left-right flow to color Doppler mapping.

Interventricular septal with minimal trabecular mulecular interventricular communication with left-right shunt to color Doppler mapping.

Closed arterial channel

LEFT CAVITIES:

Left atrium: Normal size

Left ventricle: Normal dimension. Normal myocardial thickness. Normal systolic function

RIGHT CAVITY:

Right atrium: Normal size.

Right ventricle: Normal size. Normal myocardial thickness. Normal systolic function

BASIC VESSELS:

Aorta: Normal size. Left aortic arch, unobstructed along its path.

Crowns of habitual origin.

Pulmonary artery: Main pulmonary artery and right and left pulmonary arteries of normal dimensions.

VALVAR APPLIANCES:

The cardiac valves present normal characteristics, as well as the analysis of their flow to the color flow mapping.

PERICARDIUM:

Absence of pericardial effusion.

CONCLUSION:

Persistent oval foramen

Minimum apical muscular interventricular communication.

Preserved biventricular function.
